# The Mechanobiology of Endothelial-to-Mesenchymal Transition in Cardiovascular Disease

**DOI:** 10.3389/fphys.2021.734215

**Published:** 2021-09-09

**Authors:** Shahrin Islam, Kristina I. Boström, Dino Di Carlo, Craig A. Simmons, Yin Tintut, Yucheng Yao, Jeffrey J. Hsu

**Affiliations:** ^1^Division of Cardiology, Department of Medicine, David Geffen School of Medicine at UCLA, Los Angeles, CA, United States; ^2^UCLA Molecular Biology Institute, Los Angeles, CA, United States; ^3^Veterans Affairs Greater Los Angeles Healthcare System, Los Angeles, CA, United States; ^4^Department of Bioengineering, University of California, Los Angeles, Los Angeles, CA, United States; ^5^Department of Electrical and Computer Engineering, University of California, Los Angeles, Los Angeles, CA, United States; ^6^Department of Mechanical and Industrial Engineering, University of Toronto, Toronto, ON, Canada; ^7^Institute of Biomedical Engineering, University of Toronto, Toronto, ON, Canada; ^8^Translational Biology and Engineering Program, Ted Rogers Centre for Heart Research, Toronto, ON, Canada; ^9^Department of Physiology, University of California, Los Angeles, Los Angeles, CA, United States; ^10^Department of Orthopedic Surgery, University of California, Los Angeles, Los Angeles, CA, United States

**Keywords:** endothelial-to-mesenchymal transition, mechanobiology, cardiovascular disease, endothelial, mesenchymal, biomechanical

## Abstract

Endothelial cells (ECs) lining the cardiovascular system are subjected to a highly dynamic microenvironment resulting from pulsatile pressure and circulating blood flow. Endothelial cells are remarkably sensitive to these forces, which are transduced to activate signaling pathways to maintain endothelial homeostasis and respond to changes in the environment. Aberrations in these biomechanical stresses, however, can trigger changes in endothelial cell phenotype and function. One process involved in this cellular plasticity is endothelial-to-mesenchymal transition (EndMT). As a result of EndMT, ECs lose cell-cell adhesion, alter their cytoskeletal organization, and gain increased migratory and invasive capabilities. EndMT has long been known to occur during cardiovascular development, but there is now a growing body of evidence also implicating it in many cardiovascular diseases (CVD), often associated with alterations in the cellular mechanical environment. In this review, we highlight the emerging role of shear stress, cyclic strain, matrix stiffness, and composition associated with EndMT in CVD. We first provide an overview of EndMT and context for how ECs sense, transduce, and respond to certain mechanical stimuli. We then describe the biomechanical features of EndMT and the role of mechanically driven EndMT in CVD. Finally, we indicate areas of open investigation to further elucidate the complexity of EndMT in the cardiovascular system. Understanding the mechanistic underpinnings of the mechanobiology of EndMT in CVD can provide insight into new opportunities for identification of novel diagnostic markers and therapeutic interventions.

## Introduction

**Figure 1:**
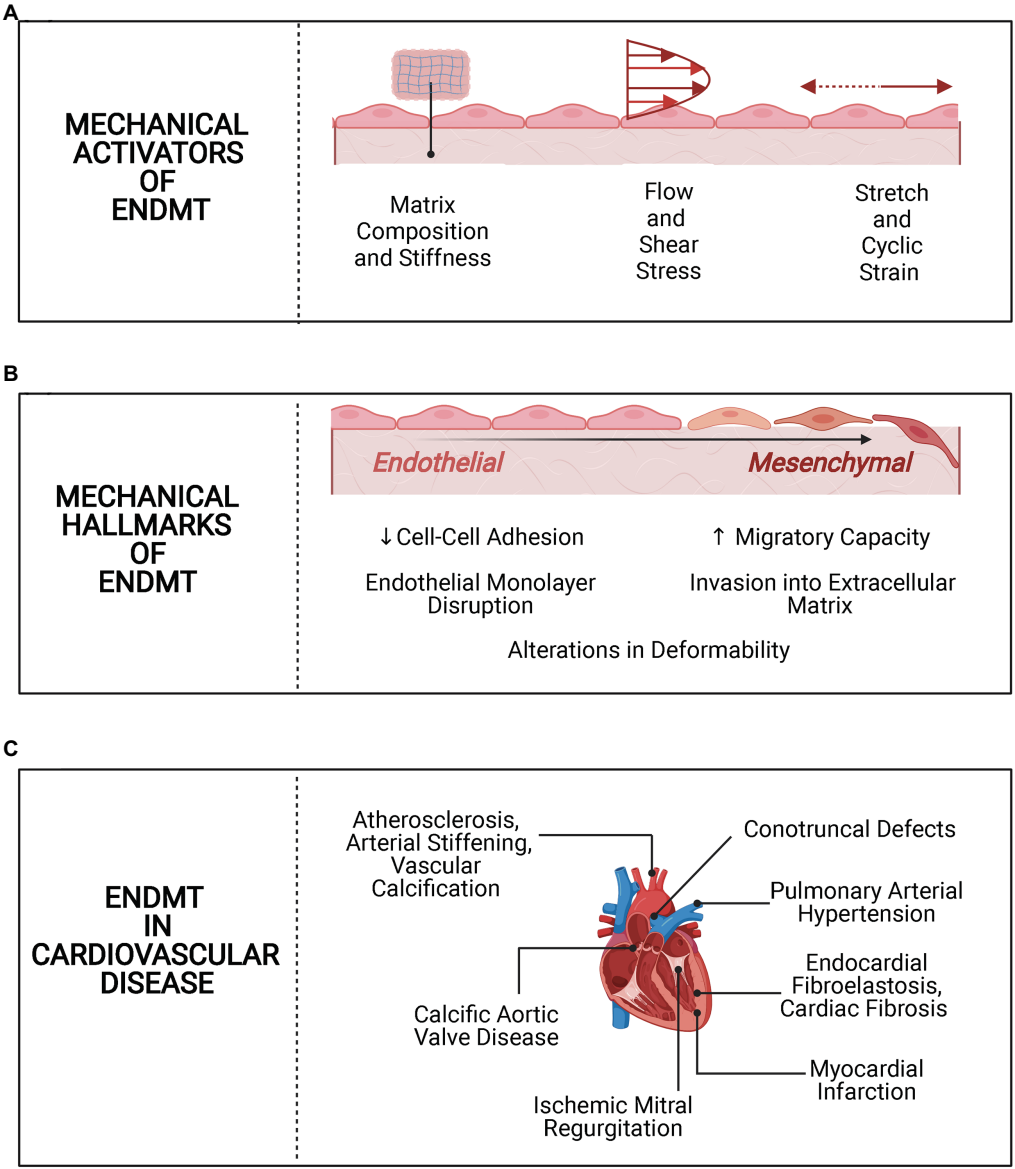
Overview of mechanobiology of endothelial-to-mesenchymal transition (EndMT) in cardiovascular disease (CVD). **(A)** Mechanical activators of EndMT. Alterations in matrix stiffness and composition, shear stress, and cyclic strain activate EndMT in endothelial cells. **(B)** Mechanical hallmarks of EndMT. Following activation of EndMT, there are mechanical changes, such as loss of cell-cell adhesion and increased migration. **(C)** EndMT in CVD. EndMT is implicated in several CVD processes, with a potential role for biomechanical alterations in each.

Endothelial cells (ECs) comprise a highly heterogeneous population of cells that line the vasculature and the endocardium in the cardiovascular system (Kalluri et al., 2019; Kalucka et al., 2020). The microenvironment of the ECs is extremely dynamic and subject to multiple mechanical stresses, such as shear stress and cyclic stretch associated with pulsatile blood flow (Chien, 2007; Charbonier et al., 2019). These stresses exist on a spectrum and can vary depending on the part of the cardiovascular system, as well as in pathologic vs. physiologic conditions. For example, shear stress under physiological conditions ranges from 10 to 70dynes/cm^2^ in the arterial system and 1 to 6dynes/cm^2^ in the venous system. In terms of cyclic strain, the physiologic range is 5 to 10% and strain above 20% is pathological and often observed in hypertension (Peng et al., 2019). In addition to shear stress and cyclic strain, the composition and mechanical properties of the extracellular matrix (ECM) are also crucial to endothelial homeostasis (Iivanainen et al., 2003; Gordon et al., 2020). Perturbations in these biomechanical elements of the microenvironment are sensed by ECs, and these signals are transduced to activate pathways that often result in phenotypic and functional changes (Kovacic et al., 2019). A notable example of this cellular plasticity is when ECs lose their endothelial features and acquire more mesenchymal-like cellular transcripts and functions through a process known as endothelial-to-mesenchymal transition (EndMT). EndMT was originally identified as form of epithelial-to-mesenchymal transition (EMT) playing a key role in the development of the cardiovascular system, but there is burgeoning evidence that it may also contribute to many cardiovascular diseases (CVD; Li et al., 2018; Kovacic et al., 2019). However, it is not yet well quite understood whether EndMT is a consequence of pathological processes involved in CVD or whether it actively contributes to CVD. While this question remains yet to be conclusively answered, the growing evidence linking EndMT to CVD has increased interest in characterizing the molecular and biomechanical changes associated with EndMT. Here, we review the mechanobiology of EndMT in CVD by describing how altered biomechanical factors activate EndMT, detailing the biomechanical hallmarks of EndMT, and summarizing the evidence of mechanically driven EndMT in various pathological contexts (Figure 1).

## Overview of Endmt

EndMT was first described in the context of heart development as important for valve formation and heart septation, which is described in detail in other reviews (Kovacic et al., 2012, 2019; von Gise and Pu, 2012; Zhang et al., 2018). Many signaling pathways are involved in the activation of EndMT, but the most extensively characterized are part of the transforming growth factor β (TGFβ)/bone morphogenetic protein (BMP) superfamily (Cooley et al., 2014; Krenning et al., 2016; Ma et al., 2020a). Other signaling pathways involved in EndMT include Notch and Wnt signaling (Cheng et al., 2013; Piera-Velazquez and Jimenez, 2019). As EMT can occur in the reverse direction through a process known as mesenchymal-to-epithelial transition (Pei et al., 2019), EndMT also has a reverse counterpart known as mesenchymal-to-endothelial transition that has been shown to contribute to neovascularization after cardiac injury (Ubil et al., 2014).

EndMT pathways can be activated through a variety of biochemical and biomechanical stimuli. Because the TGFβ signaling pathways are the most extensively studied in EndMT, many studies utilize TGFβ isoforms as the main stimulus for EndMT induction (Pérez et al., 2017). TGFβ is a multifunctional protein with highly diverse functions in embryonic development, cell proliferation, fibrosis, and regulation of the inflammatory responses. Cells vary in their responses to TGFβ as a function of their differentiation state and their cytokine milieu (Dobaczewski et al., 2011). TGFβ is often used alone as a stimulus for EndMT or in combination with other pro-inflammatory cytokines, such as tumor necrosis factor-α (TNFα) and interleukin-1β (IL1β), to investigate the impact of inflammation on EndMT in various disease models. Since many forms of CVD, including calcific aortic valve disease (CAVD), atherosclerosis, and vascular calcification, are linked to inflammation, there is increasing interest in understanding how inflammation relates to EndMT (Cho et al., 2018). Furthermore, since the cardiovascular system is dynamic and ECs experience various forms of mechanical stresses as discussed in the previous section, the potential of biomechanical alterations to induce EndMT has been investigated as well (Krenning et al., 2016). In cardiovascular pathological states linked to EndMT, such as endocardial fibroelastosis (EFE) where the role of inflammation is potentially less significant, EndMT pathways may be activated predominantly through these aberrant mechanical stresses or other factors that have yet to be well characterized. Since most forms of CVD involve both biomechanical and biochemical alterations, it is likely that there is a multipronged activation of EndMT pathways.

### Heterogeneous Responses to EndMT Stimuli

EC responses to EndMT-promoting stimuli are highly heterogeneous and are dependent on multiple factors (Pinto et al., 2016; Cho et al., 2018). For example, differences in patterns of gene expression were observed when comparing treatment with TNFα of human microvascular ECs (HMECs) vs. macrovascular human umbilical vein endothelial cells (HUVECs; Viemann et al., 2006). With regards to how arterial and venous ECs respond to treatment with EndMT-promoting biochemical stimuli, a recent study demonstrated that co-treatment of HUVECs and human pulmonary artery endothelial cells with TGFβ2 (10ng/ml) and IL1β (1ng/ml) for 7days led to EndMT induction but treatment with TGFβ2 (10ng/ml) or IL1β (1ng/ml) alone did not (Monteiro et al., 2021). In contrast, another study indicated that treatment of primary human coronary aortic endothelial cells with IL-1β (10ng/ml) is sufficient to induce EndMT after 24h (Sánchez-Duffhues et al., 2019). Like these findings, one study recently reported that treatment of HMECs with TNFα led to induction of EndMT in a dose-dependent manner from 20 to 100ng/ml at 96h (Adjuto-Saccone et al., 2021) but a previous study indicated that treatment of human intestinal microvascular endothelial cells with TNFα did not induce EndMT even after 6days (Rieder et al., 2011). These findings highlight the challenge with determining optimal methods of inducing EndMT for *in vitro* experiments due to the heterogeneity in EC responses to these stimuli that depend on multiple factors including EC subtype and dose of stimuli. A deeper characterization of the differential properties of these EC populations is needed.

Like variations in responses to treatment with inflammatory cytokines and growth factors, ECs are also influenced differentially by biomechanical stimuli, such as flow. There are multiple mechanical stresses in the cardiovascular system that are highly variable under physiological and pathological conditions. It has been shown the pattern of expression of adhesion molecules and immune cell adhesion varied not only on which vascular bed the ECs were derived from but also on whether the ECs were exposed to arterial or venous flow profiles (Methe et al., 2007). The heterogeneity in responses to biomechanical and biochemical stimuli that promote EndMT based on EC subtype is an important consideration for the study of EndMT and further work is required to better characterize the molecular mechanisms that govern these differences.

### Mechanical Regulators of EndMT, Mechanosensing, and Mechanotransduction

As discussed in the previous section, EndMT can be induced by various biochemical and biomechanical triggers in the EC microenvironment. Understanding how ECs sense and respond to mechanical forces has been a key focus of vascular biology and is covered extensively in other reviews (Chien, 2007; Krenning et al., 2016; Gordon et al., 2020). Here, we focus on how shear stress, cyclic strain and matrix stiffness, and composition regulate EndMT in CVD, and this section briefly describes how ECs sense and respond to these mechanical stresses that may be more relevant in the context of EndMT.

EC mechanosensors for detecting cyclic strain include stretch-sensitive ion channels and G-protein coupled receptors-like Gαq/11 (Naruse and Sokabe, 1993; Clark et al., 2002). Of note, uniaxial cyclic strain (10%) resulted in conformational change of stretch-sensitive calcium ion channels, such as TRPV4, of capillary ECs through integrin-ECM adhesions. This conformational change resulted in the activation of phosphatidyl inositol-3-kinase (PI3K) and downstream signaling pathways, which led to Rho and Rho-associated kinase (ROCK) mediated focal adhesion and stress fiber remodeling in a direction perpendicular to the applied tension field (Thodeti et al., 2009). This cytoskeletal reorientation is crucial for directional EC migration, which is a feature of EndMT (Lamalice et al., 2007; Clere et al., 2020).

EC responses to cyclic stretch, matrix stiffness, and shear stress are also mediated by integrins and vascular endothelial cadherin (VE-Cadherin; Schwartz and DeSimone, 2008; Tian et al., 2016). VE-Cadherin is the main cell-cell adhesion molecule in the EC monolayer. Mechanical loading of VE-Cadherin triggered cytoskeletal remodeling, which resulted in a force-dependent increase in cellular stiffness, disruption of peripheral junctions, and decrease in focal adhesions through a mechanism that involves ROCK1 and PI3K (Barry et al., 2015). Furthermore, loading of VE-cadherin also increased integrin-dependent cell contractility and disrupted cell-matrix adhesions (Andresen Eguiluz et al., 2017). Because hallmarks of EndMT include cytoskeletal remodeling and disruption of cell-cell junctions, VE-Cadherin likely plays an important role in modulating the responses of ECs to alterations in cyclic stretch, shear stress, and matrix stiffness that can initially activate EndMT pathways.

While the role of these ion channels, receptors, and integrins that are known to be mechanosensors has yet to be fully elucidated in the context of EndMT, a recent study has demonstrated that the receptor Alk5 and downstream Shc were crucial to sensing shear stress and modulating EndMT in atherosclerosis (Mehta et al., 2021). The details of this study are further discussed in Section 5.1. This is the first report of a mechanosensor and mechanotransduction pathway specifically implicated in EndMT. Further work is required to characterize how mechanical signals are detected in EndMT and specifically, which mechanotransduction pathways directly activate or suppress EndMT pathways.

### Markers of EndMT

The general description of EndMT broadly involves the loss of endothelial cellular features and gain of mesenchymal characteristics. The loss of VE-Cadherin is an important marker of EndMT progression. In addition to reduced VE-Cadherin expression, the decrease in expression of CD31, von Willebrand Factor (vWF), and endothelial nitric oxide synthase 3 also indicates the loss of the endothelial cellular phenotype, and the increase in expression of markers, such as α-smooth muscle actin (α-SMA), calponin, transgelin (SM22α), vimentin, and versican, denotes the transition to a mesenchymal state (Table 1; Dejana et al., 2017; Sánchez-Duffhues et al., 2018).

**Table 1:** Endothelial and mesenchymal markers.

**Endothelial markers**	**Mesenchymal markers**
VE-Cadherin	α-smooth muscle actin
CD31	Calponin
von Willebrand factor	Transgelin
Endothelial nitric oxide synthase 3	Versican
CD34	N-Cadherin
Tie2	Tropomyosin 1
Vascular endothelial growth factor receptor 2	Fibulin-5
	Connective tissue growth factor
	Snail
	Vimentin

However, there is a lack of consensus of an exact molecular and functional definition of EndMT (Kovacic et al., 2019). This is because the expression of these markers is time-dependent and can be variable, which poses a challenge in assessing the presence of EndMT primarily through marker expression. For example, RNA sequencing of HUVECs exposed to low, oscillatory shear stress (OSS; 0.5±5dyne/cm^2^), a potent inducer of EndMT, revealed that endothelial marker genes *NOS3*, *VWF*, and *CD34* were downregulated as early as 6h after exposure to OSS, whereas mesenchymal marker genes *CDH2*, *TPM1*, and *FBLN5* began to be upregulated around 12h (Ajami et al., 2017). In contrast, results from another similar RNA sequencing study indicated when HUVECs were subject to OSS (1±4dyne/cm^2^) for 24h, there was no significant effect on the expression of endothelial markers vWF, CD31, Tie2, vascular endothelial growth factor receptor 2, and VE-Cadherin, whereas exposure to pulsatile shear stress, which is protective against EndMT, upregulated the expression of these specific markers (Lai et al., 2018). However, this study found that OSS did increase mRNA levels of mesenchymal markers, such as N-Cadherin and connective tissue growth factor (CTGF). Interestingly, the authors found that quantitative PCR but not RNA sequencing data demonstrated the induction of vimentin by OSS. The variability in findings from these two studies underscore the challenge in utilizing expression of various markers associated with the endothelial and mesenchymal phenotypes as the primary means of assessing EndMT progression.

### Endothelial Monolayer Disruption, Invasion, and Migration During EndMT

In addition to the changes in expression of endothelial and mesenchymal markers during EndMT, there are physical changes that occur, such as cytoskeletal remodeling, loss of cell-cell adhesion, and cellular polarity (Gasparics et al., 2016). In the early stages of EndMT, a decrease in intercellular adhesion forces in the endothelial monolayer along with an increase in cellular stiffness has been reported (Sancho et al., 2017). However, parallel processes in EMT suggest that a decrease in cellular stiffness may be important for migration (Osborne et al., 2014), though this has not yet been extensively studied.

Cellular stiffness and the related property of deformability are often used to describe the mechanophenotype of a cell (Kozminsky and Sohn, 2020). For example, induction of EMT in human ovarian cancer cells demonstrated marked differences in the mechanophenotype of epithelial vs. mesenchymal cells, which correlated with EMT-mediated changes in epithelial and mesenchymal markers. The mesenchymal transformed cells were more deformable, and the parallel microfiltration technique used to assess the mechanophenotype of these transformed cells was able to determine whether cells are more mesenchymal or epithelial based on deformability alone (Qi et al., 2015). This technique along with similar microfluidic approaches that allow for monitoring of the dynamic cellular mechanophenotype may be promising label-free methods of overcoming the challenges associated with using cell surface markers as the primary means of assessing EndMT.

Following disruption of the cell-cell adhesions that constrain the ECs in the monolayer, ECs invade and migrate through the ECM, which requires degradation of the ECM and increased cellular motility. While the mechanisms of matrix invasion and migration during EndMT in CVD are not well characterized, there are elements from cancer biology that may be relevant. Tumor cells exhibit two types of cellular motility: (1) a mesenchymal pattern facilitated by matrix metalloproteinases (MMPs), which promotes degradation of the ECM and (2) an amoeboid pattern mediated by Rho/ROCK cytoskeletal contraction (Panková et al., 2010). When TGFβ2 was added to human dermal microvascular ECs, they transitioned to an intermediate mesenchymal state and upregulated the expression of MMP-2 which was proteolytically active (Kryczka et al., 2017). They also showed that this upregulation of MMP-2 was associated with an increase in the motility of the cells undergoing EndMT. Furthermore, inhibition of both MMP-2 and ROCK reduced their motility during EndMT. These results suggest that the increased motility of dermal microvascular ECs during EndMT may be a mixed subtype of the amoeboid and mesenchymal types of migration since it is dependent both on MMP-2 and Rho/ROCK.

### Paracrine Role of MMPs in EndMT

Interestingly, MMPs expressed by other cells in the vicinity of ECs may play a regulatory role in EndMT. In an animal model of myocardial infarction (MI), MMP14 expressed in macrophages induced their release of TGFβ1, which subsequently resulted in activation of SMAD2-mediated EndMT pathways in nearby ECs through paracrine signaling. MMP14 silencing *in vivo* corresponded with decreased collagen deposition and cardiac fibrosis after MI (Alonso-Herranz et al., 2020; Lim, 2021). Furthermore, with MMP14 silencing, there was reduced left ventricular dysfunction and dilatation and greater preservation of vascular supply in the cardiac tissue following ischemic injury. When MMP14 was inactivated in these macrophages *in vitro*, there was decreased EndMT. The decreased EndMT following inactivation of MMP14 in these cardiac macrophages may explain the effects of the attenuation of maladaptive sequelae of ischemic injury observed with MMP14 silencing *in vivo*. Thus, MMPs may play an important role in not only facilitating motility of ECs undergoing EndMT, but also in activating EndMT through indirect paracrine signaling.

### EndMT Continuum

**Figure 2:**
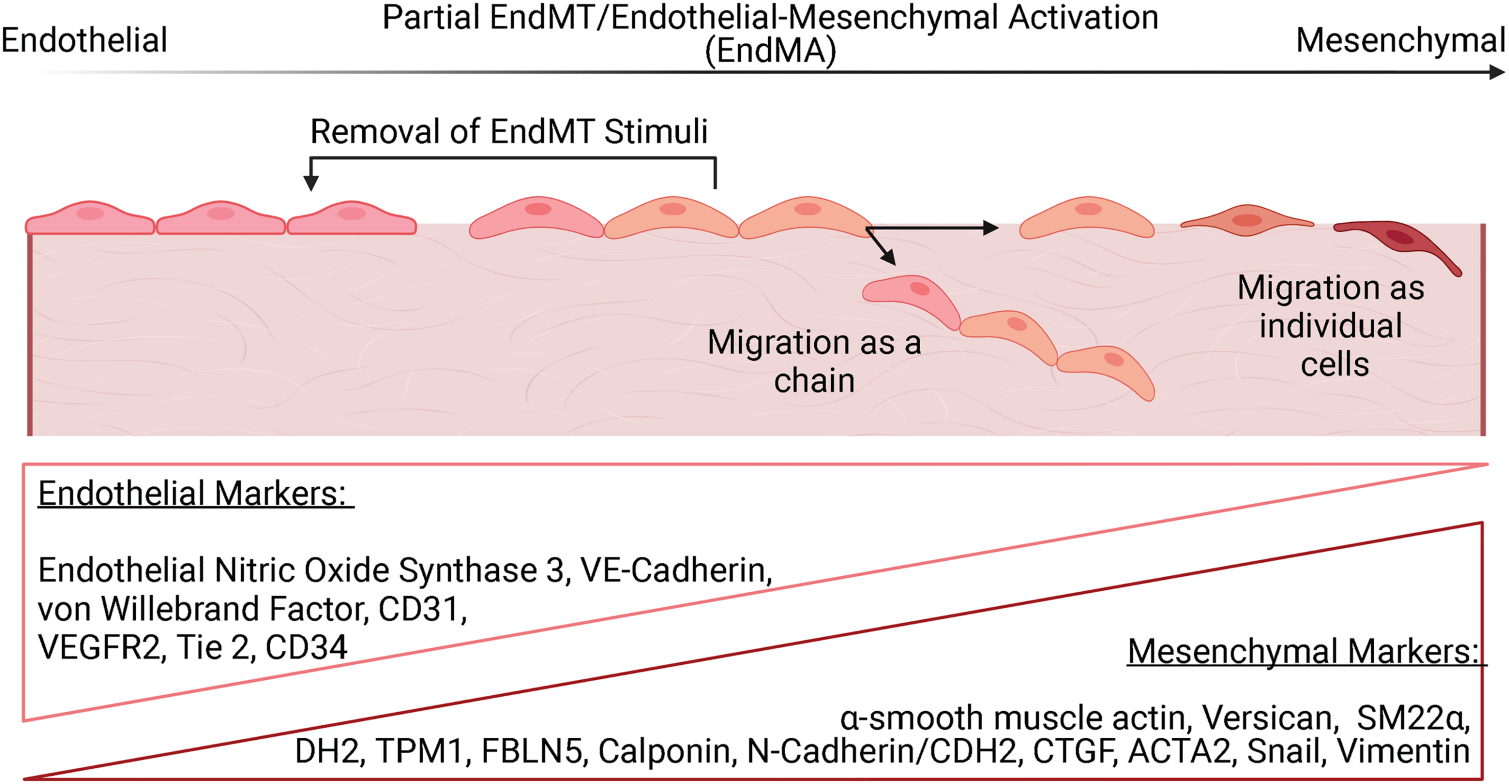
The EndMT continuum. EndMT is thought to exist on a spectrum with evidence for partial activation of EndMT that may be reversed with removal of the EndMT stimuli. Endothelial cells that have undergone partial EndMT can migrate as a chain without loss of cell-cell adhesion. ECs that undergo EndMT can also migrate as individual cells. Complete EndMT is defined as the presence of an entirely mesenchymal state. The progression of EndMT is evaluated by the monitoring the expression of various endothelial and mesenchymal markers, with ECs that have undergone partial EndMT expressing some level of both types of markers.

The phenotypic alterations associated with EndMT appear to exist on a continuum (Figure 2). In an intermediate stage of EndMT, or partial EndMT, both endothelial and mesenchymal features may be present, while complete EndMT indicates an essentially entirely mesenchymal state (Dejana and Lampugnani, 2018; Bischoff, 2019). Partial EndMT is thought to play a physiological role in angiogenesis, where ECs lack apical-basal polarity and degrade the ECM but retain some cell-cell adhesion and migrate as a chain of cells instead of as individual cells (Welch-Reardon et al., 2015). Interestingly, a recent study reported a transient mesenchymal activation of ECs along with metabolic adaptions in the first week after MI that was not sustained long term (Tombor et al., 2020, 2021). Furthermore, removal of EndMT-promoting stimuli resulted in a reversal of the mesenchymal phenotype *in vitro*. It is hypothesized that this transient EndMT allows for ECs to migrate and participate in vascularization of ischemic areas. This partial activation has been termed as endothelial-to-mesenchymal activation and opens new avenues of inquiry into how EndMT may not only be a mechanism of maladaptive remodeling following ischemic injury but could also positively influence the healing response through revascularization of ischemic tissue.

The sequence and progression of EndMT are still not yet well understood. In a pathological context of pulmonary arterial hypertension (PAH), cell lineage tracing allowed for the identification of cells in both partial and complete EndMT states with distinct patterns of marker expression and functional properties (Suzuki et al., 2017). The cells classified as partial EndMT cells demonstrated higher expression of endothelial progenitor cell markers, such as CD133/Prom1 and CD34, whereas the cells classified as complete EndMT cells did not express these markers but instead expressed Sca-1 and CD105, which are mesenchymal stem cell markers. Furthermore, these complete EndMT cells exhibited higher proliferative and migratory capacity, as well as an additional role in promoting the proliferation of non-endothelium-derived mesenchymal cells through paracrine signaling, suggesting both direct and indirect roles in the pathogenesis of PAH. Though there was coexistence of cells in both partial and complete EndMT states, it was not clear whether cells had to progress through the partial EndMT state to reach the complete EndMT state. EndMT is a complex and multifaceted phenomenon with multiple activators and signaling pathways involved, and the progression of EndMT requires further elucidation.

## Valve Development and Pathology

EndMT is crucial to the formation of the atrioventricular valves and the outflow tract (OFT). Abnormalities in OFT development can result in congenital heart diseases (CHD), such as conotruncal defects (CTD). In patients with CTD, SOX7 mutations have been identified and additional *in vitro* studies demonstrated that SOX7 mutations can lead to impaired EndMT *via* regulation of VE-Cadherin, indicating that the inhibition of EndMT during development may contribute to CTD (Jiang et al., 2021).

There are important mechanical forces that regulate EndMT in normal OFT development (Hove et al., 2003; Bartman et al., 2004; Kalogirou et al., 2014). Aberrations in contractile and hemodynamic forces lead to abnormalities in OFT development that may also contribute to CHD. During the formation of the OFT, increases in wall shear stress resulted in greater Notch1b signaling and EndMT, which then led to ventriculobulbar (VB) valve hyperplasia. In contrast, decreases in contractile forces reduced Notch1b signaling and EndMT, which subsequently resulted in VB valve underdevelopment (Hsu et al., 2019).

In addition to contributing to abnormalities of valve development associated with CHD, EndMT is also involved in other pathological settings of the valves. The following sections describe the role of EndMT in CAVD and ischemic mitral regurgitation (IMR) and how various types of biomechanical alterations can regulate EndMT of valvular ECs.

### Calcific Aortic Valve Disease

Calcific aortic valve disease is the third most prevalent cause of CVD (Yutzey et al., 2014). The pathophysiological features of CAVD include aberrant collagen orientation, leaflet thickening, and fibrosis, which can result in hemodynamic abnormalities eventually necessitating valve replacement. Additionally, an active area of investigation is the role of inflammation as one of the underlying mechanisms that drives the initiation and progression of valvular calcification in CAVD (Lim et al., 2016). While initially believed to be a passive, degenerative process, there is now more evidence indicating that valvular calcification is an active and dynamically regulated process with multiple cell types playing a role (Ma et al., 2020b). It is thought that dysregulation of EndMT causes differentiation of resident cells into the osteogenic and fibrotic cell types that result in the calcified, stiff aortic valve leaflets observed in CAVD.

**Figure 3:**
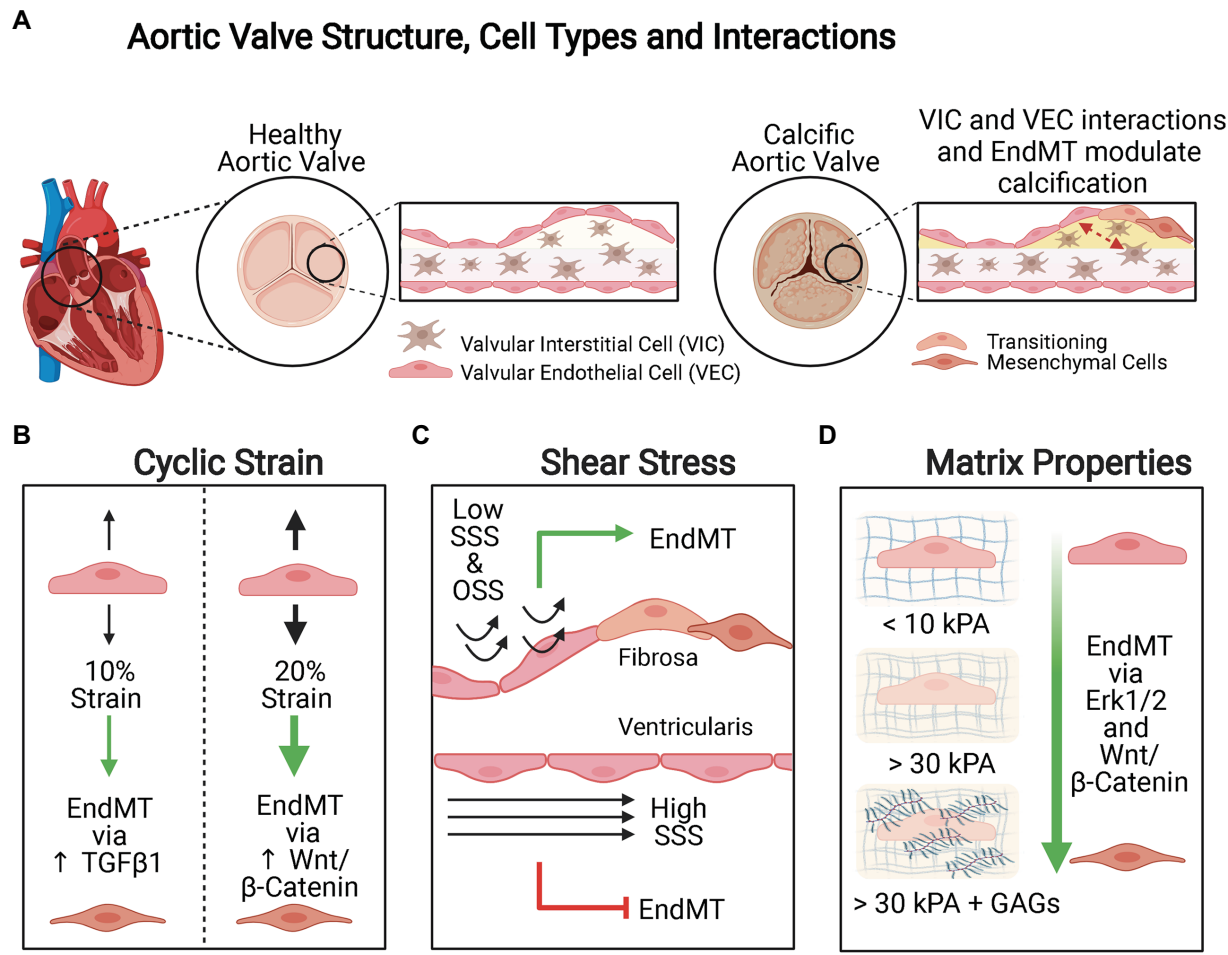
Mechanical modulation of EndMT in valvular endothelial cells (VECs). **(A)** Aortic valve structure, cell types, and interactions. The aortic valve is a trilaminar structure with valvular interstitial cells (VICs) sandwiched in between two layers of VECs. Interactions between the VICs and VECs and EndMT are both important in valvular calcification. **(B)** Cyclic strain. Cyclic strain induces greater extent of EndMT when applied orthogonally to the alignment of the valve endothelium and the pathways activated are magnitude dependent. When VECs are subject to 10% cyclic strain, EndMT is activated *via* TGFβ1 signaling, whereas 20% cyclic strain promotes EndMT *via* Wnt/β-Catenin signaling. **(C)** Shear stress. Low steady shear stress (SSS) and oscillatory shear stress (OSS) promote EndMT, whereas high SSS is protective against EndMT. Calcification is more common on the fibrosa side where VECs are exposed to low SSS or OSS than on the ventricularis side where VECs are subject to high SSS. **(D)** Matrix composition and stiffness. Increasing matrix stiffness and increased glycosaminoglycans (GAGs) content promote EndMT.

The aortic valve leaflet has a trilaminar structure and is broadly composed of two types of cells: valvular endothelial cells (VECs) and valvular interstitial cells (VICs). Interactions between these cells contribute to the formation of calcific lesions (Figure 3A). Indirect coculture of VECs and VICs in hydrogels with equi-biaxial mechanical constraint and supplementation of osteogenic differentiation factors demonstrated that VECs promote enhanced calcification and pathological remodeling of VICs *via* EndMT and osteogenic differentiation when compared to hydrogels seeded with VICs only (Gee et al., 2021). Furthermore, when porcine aortic VECs were treated with TNFα and cultured on a three-dimensional system that allowed transforming and non-transforming cells to be independently isolated and characterized, both non-transformed cells that maintained control levels of endothelial VE-cadherin and eNOS and transformed cells that lost these endothelial characteristics and had increased expression of α-SMA were identified. These results suggest that only a certain subset of VECs may be susceptible to undergoing EndMT under conditions of inflammation and that interactions between non-transformed and transformed cells may also be an important factor in CAVD pathogenesis in addition to interactions between VECs and VICs (Farrar and Butcher, 2014).

While these studies highlight how different cell types interact in the context of EndMT in CAVD, the lineages of the cell types present in calcific lesions in CAVD was not explored. However, a recent single-cell RNA sequencing study detailing a transcriptomic atlas of human aortic valves from both healthy and CAVD patient samples not only demonstrated remarkable cellular heterogeneity in the aortic valve leaflets but also provided more direct evidence of EndMT as the process that leads to the differentiation of resident VICs and VECs into newly identified valve-derived stromal cells that are prominent in only the CAVD samples (Xu et al., 2020). The pseudotime trajectory analysis along with other cellular localization experiments performed in this study suggests that EndMT was actively involved in the thickening of the calcified aortic valve leaflets, rather than a consequence of the CAVD process.

### Mechanical Modulation of Valvular EndMT

While single-cell RNA sequencing allowed for the identification of distinct subpopulations of cells and suggested that EndMT contributes to this cellular heterogeneity in human CAVD, it did not provide mechanistic insight into what initiates and promotes EndMT of the resident VECs and differentiation of VICs. This is not yet well understood, but there are studies that have characterized the effect of varying cyclic strain, shear stress, matrix stiffness, cell-cell, and cell-matrix interactions on VECs, VICs, and EndMT (Figure 3). These studies indicated that VECs can undergo EndMT under a variety of conditions, and it is likely that a combination of these factors play a role in promoting EndMT that ultimately drive the initiation and progression of CAVD. It is important to note that many of these studies aim to elucidate the impact of modulating various mechanical parameters on EndMT of VECs in general. Therefore, the findings may be applicable to other valvular pathologies that involve EndMT beyond CAVD.

Cyclic strain induces EndMT of VECs in a magnitude and direction-dependent manner (Figure 3B). When VECs were subjected to low strain (10%) representing physiologic conditions, there was increased expression of the mesenchymal marker α-SMA and downregulation of endothelial markers VE-Cadherin and CD31, in addition to increased TGFβ1 signaling. Under conditions of high strain (20%) mimicking pathological conditions, the expression of these markers was modulated *via* increased Wnt/β-catenin signaling (Balachandran et al., 2011). Furthermore, the authors found that cyclic strain applied orthogonally to the alignment of the valve endothelium resulted in more pronounced disruption of cellular microarchitecture, more extensive EndMT, increased contractility in the presence of endothelin-1, and greater basal mechanical tone.

In addition to cyclic strain, VECs also experience different hemodynamic stresses due to pulsatile blood flow (Figure 3C). VECs subjected to low steady shear stress (SSS; 2dyne/cm^2^) and OSS demonstrated increased expression of EndMT-related markers Snail and α-SMA, TGFβ1 along with inflammation-related markers ICAM1 and NF-κB1, when compared to cells exposed to high SSS (10 and 20dyne/cm^2^) or static conditions (Mahler et al., 2014). Furthermore, VECs exposed to low SSS condition demonstrated increased matrix invasion. The cells also elongated and aligned perpendicularly to the laminar SSS but not the OSS. These results were consistent with the observation of EndMT on the fibrosa side (facing the aorta) where cells experience low OSS and thought to be the initiating site of inflammation and calcification (Mohler, 2004), whereas EndMT is not observed on ventricularis side (facing the left ventricle) which is exposed to high SSS (Mahler et al., 2013). While these conditions of shear stress utilized may not recapitulate physiological or pathophysiological conditions precisely, these results nonetheless highlight why low SSS or OSS promotes inflammation and EndMT, whereas high SSS is protective against EndMT of VECs.

The components and mechanical properties of the ECM of VECs are also important factors that modulate EndMT (Figure 3D). In healthy porcine aortic valve tissue, the fibrosa side has been reported to be stiffer with effective Young’s moduli >3kPa, whereas the ventricularis side is softer with effective Young’s moduli <0.5kPa (Zhao et al., 2011). When healthy porcine aortic VECs were seeded into stiffer gel matrices (~37–50kPa), they demonstrated greater expression of EndMT-related markers, such as α-SMA, than cells that were seeded into less stiff gel matrices (~2–5kPa). This suggests that increasing matrix stiffness alone can induce mesenchymal transformation of healthy VECs (Dahal et al., 2017). Furthermore, they found that the presence of glycosaminoglycans (GAGs) in the ECM increased the expression of EndMT-related markers when comparing cells seeded into matrices of similar stiffness. GAGs weakened the cell-ECM adhesion strength, altered ECM binding, and influenced production of collagen I and GAGs by the newly transitioned mesenchymal cells.

The effects of matrix stiffness on EndMT of VECs involve the ERK1/2 and Wnt/β-catenin signaling pathways. When VECs seeded in collagen gels with and without GAGs were treated with the ERK1/2 inhibitor U0126, there was a significant downregulation of EndMT markers and diminished cell invasion into the matrix (Dahal et al., 2017). For VECs seeded into silicone Sylgard 527 substrates, the addition of TGFβ1 preferentially promoted EndMT on VECs seeded into stiffer substrates as demonstrated by enhanced expression mesenchymal α-SMA (Zhong et al., 2018). In these cells, the authors also observed greater β-catenin nuclear translocation. However, treatment of VECs with TGFβ1 and endostatin, which degrades β-catenin, did not reduce disruption of the endothelial monolayer, morphological changes related to EndMT, or loss of VE-cadherin localization to the cell membrane but did significantly reduce the expression of α-SMA. This suggests that β-catenin signaling does not play a role in initiating EndMT in VECs but is required for TGFβ1 mediated transformation of VECs to myofibroblasts.

### Ischemic Mitral Regurgitation

**Figure 4:**
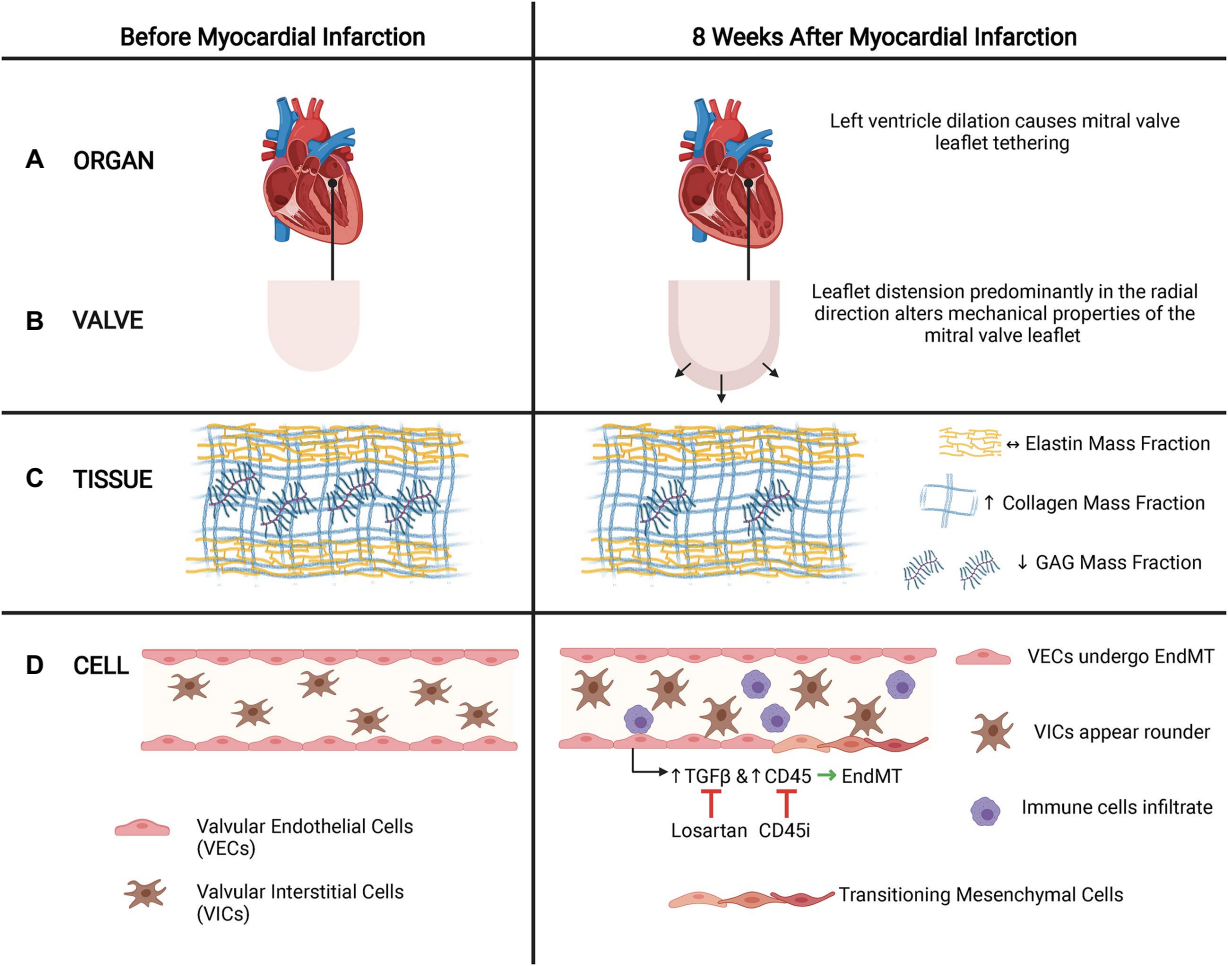
Leaflet tethering and EndMT in ischemic mitral regurgitation (IMR). **(A)**. Organ. After a myocardial infarction, the heart often dilates, which causes tethering of the mitral valves that can lead to IMR. **(B)** Valve. Tethering distends the leaflet predominantly in the radial direction. **(C)** Tissue. Leaflet tethering alters the mitral valve matrix composition by increasing the collagen mass fraction and decreasing the GAG mass fraction. However, the elastic mass fraction does not change. **(D)** Cell. These biomechanical alterations of the valve leaflet may trigger EndMT of the mitral valve endothelial cells *via* mechanisms that involve TGFβ signaling and CD45. Inhibiting TGFβ with losartan or CD45 with a CD45 inhibitor reduces EndMT and associated maladaptive responses observed in IMR. In addition to EndMT of VECs, VICs also appear rounder and immune cells may infiltrate. (Adapted with permission from Howsmon et al., 2020).

Ischemic mitral regurgitation (IMR) is a prevalent cause of valvular disease and is a consequence of leaflet tethering following papillary displacement due to left ventricle dilation after MI (Marwick et al., 2009). Tethering of mitral valve leaflets 8weeks after MI resulted in notable permanent radial deformation of the leaflets, which led to a complete loss of mechanical anisotropy, significant decrease in radial peak strain, and an increase in the collagen and GAG mass fraction (Howsmon et al., 2020; Figure 4). Furthermore, tethered leaflets also stained positively for EndMT-related markers, with more extensive presence of EndMT when there was MI in addition to leaflet tethering (Dal-Bianco et al., 2009, 2016), suggesting that these altered biomechanical stresses may trigger EndMT of mitral VECs.

In a model of leaflet tethering with MI, treatment with losartan reduced the fibrosis and thickening of the mitral valve, suggesting that losartan, which has been associated with inhibition of TGFβ signaling, may partially limit the EndMT-mediated maladaptive response of mitral VECs after MI that eventually leads to IMR (Bartko et al., 2017). Their subsequent studies of how mitral VECs respond in post-infarct conditions revealed an increase in expression of CD45, a protein tyrosine phosphatase, in addition to EndMT-related markers α-SMA and VE-cadherin in mitral valve leaflets. This was also replicated *in vitro* when addition of TGFβ to mitral VECs induced increased expression of CD45 and α-SMA (Bischoff et al., 2016). Inhibiting CD45 reduced expression of α-SMA, indicating that CD45 may regulate EndMT involved in fibrosis and thickening of mitral valve leaflets in IMR. Targeting various aspects of the EndMT pathways at either the level of TGFβ or CD45 can potentially be a promising method of inhibiting the maladaptive response of mitral VECs to altered mechanical stresses that are associated with the development and progression of IMR. Alternatively, surgical approaches, such as plication of the infarct region that decrease tethering distance and subsequently reduce stretch (Liel-Cohen et al., 2000), may prevent the induction of EndMT pathways that result in the progressive fibrosing and thickening of the mitral valve leaflets.

## Endocardial Fibroelastosis and Cardiac Fibrosis

Mitral regurgitation and other valvular pathologies, such as mitral stenosis and aortic insufficiency, can lead to the formation of flow jets (Maganti et al., 2010) that alter the hemodynamic stresses experienced by ECs lining the endocardium, which may subsequently promote EndMT of endocardial ECs. In samples from patients with flow disturbances due to stenotic or incompetent valves, there was evidence of EFE in regions where there was exposure to disturbed flow (d-flow) patterns (Weixler et al., 2020). Further staining for EndMT-related markers in those areas demonstrated increased expression of α-SMA, suggesting that EFE may be due to EndMT induced by d-flow patterns and altered shear stress from the valvular defects. In patients with hypoplastic left heart syndrome, EFE progression occurred despite surgical resection if the valvular defect was not addressed. The evidence of EndMT in EFE is consistent with other studies that have correlated increased EndMT in EFE *via* a mechanism that in part involved transcriptional suppression of BMP5 and BMP7 due to abnormal promoter methylation (Xu et al., 2015). While these studies linking EndMT to EFE have increased interest in targeting EndMT as a therapeutic approach, other studies suggest that the fibroblasts present in EFE tissues may arise from embryonic EMT rather than EndMT (Zhang et al., 2017).

EFE is a subtype of cardiac fibrosis. The role of EndMT in cardiac fibrosis more broadly has been debated. Early studies indicated that the majority of fibroblasts that appeared after cardiac injury was derived from EndMT (Zeisberg et al., 2007) and subsequent ones contrarily suggested that fibroblasts did not arise singularly from one specific differentiation program (Ali et al., 2014; Fu et al., 2018). The extent to which EndMT contributes to the cardiac fibrosis and the mechanical regulators of these processes merit further investigation.

## Vascular Disease: Atherosclerosis, Vascular Calcification, and Arterial Stiffening

Broadly, atherosclerosis and arterial stiffening are distinct but interrelated pathologies in which EndMT may play a role. The following sections highlight emerging evidence indicating the presence of EndMT in these vascular pathologies.

### Atherosclerosis and Vascular Calcification

Atherosclerosis is the most prevalent underlying cause of many types of CVD. Broadly, atherosclerosis refers to the progressive accumulation of fat and fibrous content in the intima of the arteries. Over time, the atherosclerotic plaques become more fibrous and undergo calcification (Mohler, 2004; Chen et al., 2020). The plaques often are unstable, and their rupture leads to the formation of thrombi that can occlude the vasculature and lead to acute ischemia. In advanced atherosclerosis, the plaque may cause hemodynamic disturbances by narrowing the arterial lumen. This narrowing impedes blood blow and can also result in ischemia (Libby et al., 2019).

The mechanisms underlying the pathophysiology of atherosclerosis involve multiple cell types, including ECs, fibroblasts, smooth muscle cells, macrophages, and other immune cells. Due to the high prevalence and potential serious consequences of atherosclerosis, there has been immense interest in understanding not only how these various cell types contribute to pathologic progression but also their differentiation trajectories, as well. EndMT is thought to be one key process that leads to the differentiation of resident ECs into pro-atherogenic cells (Chen et al., 2020). Early studies indicated a potential role of EndMT in atherosclerosis by demonstrating the presence of EndMT-related markers in human atherosclerotic lesions and identified hemodynamic alterations as a crucial mediator of EndMT *via* fibroblast growth factor receptor 1 signaling (Chen et al., 2015). Recent single-cell transcriptomic analyses detailing the microanatomy of advanced human atherosclerotic plaques have substantiated the evidence that EndMT may play an active role in the development and progression of atherosclerosis by identifying distinct clusters of ECs in atherosclerotic lesions that demonstrated both smooth muscle and endothelial cellular features (Depuydt et al., 2020).

**Figure 5:**
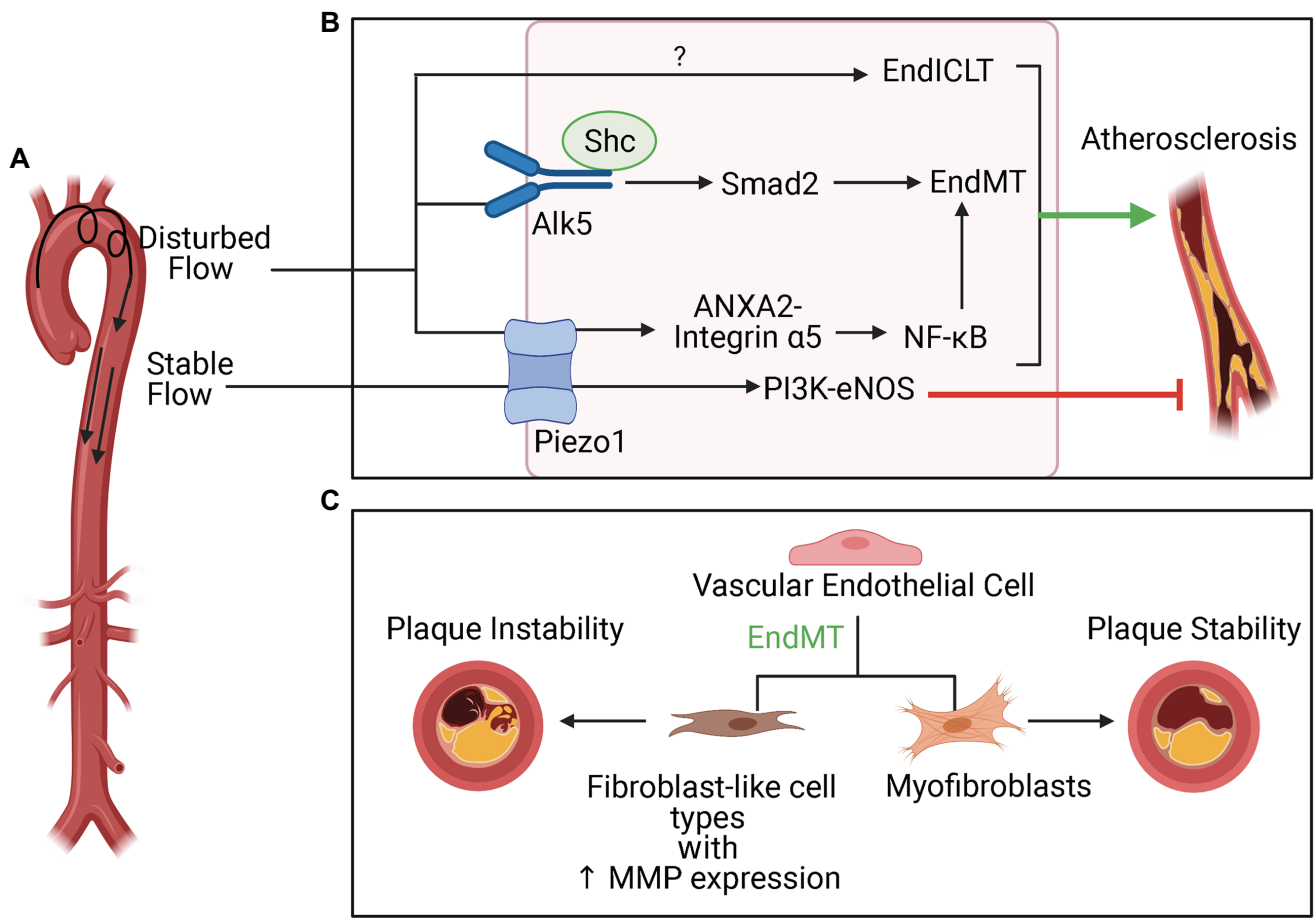
Disturbed flow (d-flow), EndMT, and plaque stability in atherosclerosis. **(A)** d-flow that occurs at the lesser curvature of the aortic arch is atherogenic, whereas stable flow typically presents in the descending aorta is atheroprotective (EndICLT). **(B)** The mechanosensor Piezo1 is crucial to the differential responses to these hemodynamic profiles. In response to d-flow, Piezo1 activates the NF-κB pathway, which is known to be important to inflammation-mediated EndMT. In addition to EndMT, d-flow also activates of endothelial-to-immune-cell-like transition. The mechanosensor Alk5 and associated protein Shc have been directly linked to activation of Smad-2 mediated EndMT pathways in response to d-flow in atherosclerosis. **(C)** EndMT can contribute to both plaque instability and plaque stability depending on whether EndMT results in transition of ECs to fibroblast-like cell types with increased matrix metalloproteinase expression or to myofibroblasts.

Atherosclerotic lesions tend to initiate in areas of the vasculature exposed to d-flow, which is a hemodynamic profile characterized by low, OSS (Dai et al., 2004; Figure 5). D-flow is linked to EC dysfunction and an atheroprone phenotype. In contrast, stable flow (s-flow) characterized by unidirectional, high laminar shear stress that can either be pulsatile or nonpulsatile is thought to be crucial to EC homeostasis and atheroprotection. Many sensors and pathways involved in transducing hemodynamic signals in ECs have been identified. Of note, Piezo1 is a mechanosensor that modulates responses to s-flow and d-flow differentially (Demos et al., 2020). Piezo1 is a nonselective cation channel that is permeable to calcium. In response to s-flow, Piezo1 activated the PI3K-eNOS pathway involved in atheroprotection. In contrast, d-flow resulted in the activation of the NF-κB pathway associated with a pro-atherogenic phenotype. This pathway involved Piezo 1-dependent activation of annexin A2, which bound integrin α5 and led to subsequent translocation of the complex to lipid rafts (Zhang et al., 2020). There integrin α5 was activated and led to the stimulation of the NF-κB pathway, which resulted in inflammation and formation of atherosclerotic plaques. Because NF-κB is a pathway that is known to be important to inflammation-mediated EndMT (Maleszewska et al., 2013; Pérez et al., 2017), Piezo-1 may play an important mechanosensory role in modulating d-flow induced EndMT in atherosclerosis.

While the role of Piezo1 in EndMT specifically is not yet known, Alk5 is a receptor that has been recently identified as crucial to modulating EndMT induced by d-flow in atherosclerosis (Mehta et al., 2021, 5). Results from this study demonstrated that depletion of Alk5 reduces shear stress-induced EndMT signaling, which involves activation of Smad2 and downstream upregulation of mesenchymal and ECM genes. Alk5 associated with Shc in response to shear stress and modulated EndMT *in vitro* and *in vivo*. Furthermore, deletion of Shc reduced atherosclerotic plaque formation in areas of d-flow *in vivo*. These findings highlight Alk5 and Shc as crucial to mediating the EndMT response in areas of d-flow prone to atherosclerotic plaque formation. Interestingly, tensional force and reconstitution experiments identified Alk5 as mechanosensor unique and sufficient for activation of Smad2 with functions independent of mechanosensors, such as PlxnD1, which plays a role in d-flow induced atherosclerosis (Mehta et al., 2020), and PECAM-1. While these experiments indicated that Alk5 leads to activation of Smad2 which is crucial to a pathway involved in EndMT even in cells that do not express PlxnD1 and PECAM-1, these other mechanosensors may still play a role in activation of other EndMT pathways independent of Smad2. Further work is required to characterize the role of other shear-sensitive mechanosensors in EndMT in atherosclerosis.

In regions exposed to d-flow, the transcription factor TWIST was expressed preferentially and contributed to activating pathways involved in inflammation, EC proliferation, and EndMT (Mahmoud et al., 2016). Downstream of TWIST, EndMT induced by low shear stress associated with d-flow involved the transcription factor Snail (Mahmoud et al., 2017). In contrast, high laminar shear stress associated with s-flow was atheroprotective through the activation of Krüppel-like factor 2 and 5 *via* Erk5 signaling (Moonen et al., 2015). EndMT induced by d-flow was inhibited *via* activation of Erk5.

In addition to promoting atherogenesis, EndMT also contributed to the calcification of atherosclerotic lesions through a mechanism partially mediated by sex-determining region Y-box 2 (SOX2; Boström et al., 2016). The role of inflammation and EndMT in calcification was also investigated by treating human aortic endothelial cells (HAECs) with TNFα and IL-1β to induce EndMT. These transformed cells then underwent osteogenic differentiation in response to BM9. It was found that BMPR2 downregulation and JNK activation were key events in BMP-9 induced mineralization (Sánchez-Duffhues et al., 2019). Activation of BMP signaling also induced the expression of serine proteases, such as elastases and kallikreins, which then triggered EndMT of HAECs. Activation of BMP signaling also induced the expression of serine proteases, such as elastases and kallikreins, which then triggered EndMT of HAECs through a pathway that involved mutual regulation of SOX2 and TWIST (Yao et al., 2015). Furthermore, endothelial-specific deletion of SOX2 *in vivo* decreased expression of these serine proteases, EndMT, and aortic calcification, suggesting the crucial role of SOX2 in vascular calcification.

Calcified atherosclerotic plaques can be either unstable or stable, and considerable effort has gone into understanding the mechanisms behind plaque rupture vulnerability (Bentzon et al., 2014). Atherosclerotic plaques that are particularly unstable and more prone to rupture demonstrated greater extent of EndMT (Figure 5), which caused ECs to differentiate into fibroblast-like cell types with increased expression of MMPs that contributed to plaque instability (Evrard et al., 2016). On the other hand, EndMT may also contribute to plaque stability. EndMT resulted in the differentiation of ECs into myofibroblast-like cells that populated the thick fibrous cap associated with stable atherosclerotic plaques (Newman et al., 2021).

Along with activation of EndMT pathways, d-flow can also activate pathways that lead ECs to differentiate into other pro-atherogenic cell types. Single-cell RNA sequencing of ECs exposed to d-flow led to the identification of multiple pro-atherogenic cell types, including pro-inflammatory cells, hematopoietic stem cells, endothelial progenitor cells, and immune cell-like phenotypes (Andueza et al., 2020). This remarkable plasticity of ECs in response to d-flow demonstrates how the development and progression of atherosclerotic plaques may involve a complex interplay between multiple pro-inflammatory cell types that arise when the vasculature is exposed to certain hemodynamic stresses. Moreover, the results from this study indicate that certain ECs are more susceptible to undergoing EndMT while others undergo endothelial-to-immune-cell-like transition. Understanding the factors that govern the fate of ECs and determine what type of transition is activated in response to d-flow may be an important consideration to develop targeted therapeutic approaches for atherosclerosis.

### Arterial Stiffening

D-flow can also lead to arterial stiffening in the absence of atherosclerosis through a mechanism that partially involved the stimulation of profibrotic genes by thrombospondin-1 (Kim Chan Kim et al., 2017). Certain ECs, such as HUVECs, responded to increased stiffening by upregulating expression of TGF-β2 and enhancing cell-matrix traction stresses (Bastounis et al., 2019). This increase in TGFβ2 signaling may result in the activation of EndMT pathways, which contribute to EC dysfunction. EndMT as a response to increased stiffness is partially mediated by Glypican 1, a core protein in the glycocalyx layer. HUVECs cultured on polyacrylamide (PA) gels of substrate stiffness 10kPa, which approximates subendothelial stiffness of aged, unhealthy arteries, demonstrated decreased glycocalyx expression, and increased expression of EndMT markers when compared to cells cultured on softer PA gels (2.5kPa), which approximates the subendothelial stiffness of young, healthy arteries. The EndMT-related markers that are upregulated are N-cadherin, α-SMA, and Snail, but interestingly, there was no difference in the expression of CD31 on cells cultured on soft vs. stiff gels, indicating that increasing stiffness may only trigger a partial EndMT. When Glypican 1 was silenced in cells are cultured on the 2.5kPa gels using siRNA, there was an increase in the expression in the EndMT-related markers N-cadherin, α-SMA, and Snail. In a model of age-mediated stiffness, there was reduced expression of Glypican 1 and greater EC dysfunction in older mice compared to young mice. Furthermore, Glypican 1 gene deletion exacerbated EC dysfunction in young mice but not aged mice (Mahmoud et al., 2020). These results suggest that the glycocalyx layer of ECs plays an important protective role against EndMT associated with increasing stiffness of the endothelial microenvironment present in age-mediated arterial stiffening.

## Conclusion

Since the identification of EndMT as a distinct form of EMT crucial for the physiological development of the cardiovascular system, there has been a growing interest in also understanding its role in various CVD processes. Because ECs in the cardiovascular system are highly sensitive to mechanical stresses that are often distinctly different under physiologic and pathologic conditions, elucidating how variations in biomechanical properties of the EC microenvironment regulate EndMT has been a key focus area. Early studies relied on *in vitro* assessments, animal models, and staining of human pathological specimens to provide evidence linking mechanically driven EndMT to CVD, though it is still quite not yet well understood whether EndMT drives CVD pathogenesis or whether it is a consequence of the pathological progression. The advent of single-cell RNA sequencing analyses has provided more insight into the differentiation trajectory of ECs in samples from patients with CVD. These studies indicate that EndMT may indeed drive the progression of the pathological mechanisms underlying CVD and actively contribute to CVD. In addition to substantiating evidence indicating that EndMT actively contributes to CVD, transcriptomic studies have also increased our understanding of the remarkable plasticity of ECs in response to aberrations in their microenvironment and the time-dependent course of these phenotypic transitions. While some studies indicate that EndMT can exist in a partial and reversible state, the permanence of epigenetic and genetic changes associated with EndMT has yet to be well characterized. Furthermore, there is also now burgeoning evidence that EndMT may have beneficial effects in pathological settings, such as contributing to revascularization after ischemic injury and atherosclerotic plaque stability. This evidence requires careful consideration of how to modulate EndMT in pathological settings as a therapeutic approach. The factors governing the plasticity of ECs and the maladaptive vs. beneficial effects of EndMT in CVD still require further elucidation and may be important for ultimately determining ideal therapeutic targets in CVD.

Nonetheless, it is evident that mechanical factors are important in the activation and regulation of EndMT. In this review, we summarized literature that linked alterations in shear stress, cyclic strain and stretch, and matrix stiffness to EndMT in CAVD, atherosclerosis, IMR, EFE, and arterial stiffening. While the mechanical stresses in the cardiovascular system are highly diverse and are often difficult to recapitulate exactly experimentally, and specific sequential progression of these pathways has yet to be fully detailed, addressing the aberrant mechanical forces that are linked to EndMT may be a promising therapeutic approach. For example, devices or surgical procedures that address the valvular defects that alter hemodynamics in patients with EFE may reduce the extent of EndMT that contributes to EFE. Similarly, reducing leaflet tethering in IMR through plication of the infarct region can reduce the stretch that activates EndMT pathways that contribute to IMR. Furthermore, there have been continued improvements and clinical utilization of computational fluid dynamic modeling to obtain patient-specific wall shear stress measurements in vascular beds, such as the coronary arteries, potentially identifying individuals at increased risk of atherosclerotic disease progression (Gijsen et al., 2019). Integration of such hemodynamic assessments along with a deeper understanding of the signaling pathways that mediate pathological responses to shear (e.g., Alk5) may help to identify therapeutic targets that are particularly effective at preventing or reversing disease progression in these at-risk patient groups.

In contrast to the amount of literature linking alterations in biomechanical stresses to the presence of EndMT in various pathological states, there is a dearth of studies detailing the mechanics associated with the key features of EndMT, such as decreased cell-cell adhesion, increased contractility and motility, and greater migratory and invasive capabilities. These features involve multiscale changes from the subcellular to tissue levels that lead to functional differences of importance in disease pathology, and further investigation of the mechanisms underlying the hallmarks of EndMT will enhance our understanding of EndMT and may help address the fact that there currently is no exact functional and molecular definition of EndMT. Characterization of biomechanical changes that ECs undergo during EndMT may also be a means of finding a mechanical biomarker that can potentially be used for the development of diagnostic tools. Indeed, mechanophenotyping technologies have shown promise by demonstrating the ability to identify cells of varying phenotypes in heterogeneous tumors (Kozminsky and Sohn, 2020). For example, real time deformability cytometry, which utilizes the physical property of cellular deformability to identify cells with increased pathological potential in heterogeneous tumors, has been proposed as a tool for cytopathology that can enable clinical decision making for screening, staging, and monitoring of treatment efficacy (Di Carlo, 2012). Further, deformability cytometry can help to identify metastatic potential of breast and prostate cancer cells (Ahmmed et al., 2018). Targeting the more metastatic cells may be a more effective means of treatment, and microfluidic technologies not only enable isolation and characterization of more pathogenic cell types from heterogeneous samples but can also allow for screening of therapeutics. While these approaches have not been attempted in the realm of CVD, it is possible that only certain subtypes of ECs are actively contributing to pathological progression in EndMT, and these tools offer a novel way of identifying and isolating these cells by their biomechanical properties.

In addition to lack of this definition of EndMT, there are challenges in tracking EndMT progression due to the variability in marker expression. Furthermore, it is not well understood whether there is differential activation of EndMT dependent on EC subtype and type of EndMT stimuli. More broadly, the role of EndMT in homeostatic conditions beyond development and disease is not well characterized. As the evidence for the role of EndMT in CVD continues to mount, it will become increasingly important to explore these fundamental questions regarding EC plasticity and EndMT mechanobiology. Due to the purported involvement of EndMT in many types of CVD, further investigation into these open avenues of inquiry may lead to the development of novel therapeutics and technologies that could improve CVD diagnosis and treatment.

## Author Contributions

SI: conceptualization, methodology, investigation, and writing of manuscript. KB, DD, CS, YT, and YY: methodology and investigation. JH: conceptualization, investigation, writing of manuscript, supervision, and project administration. All authors contributed to the article and approved the submitted version.

## Funding

Funding for this work was provided in part by the NIH/NHLBI [grant numbers 1K08HL151961 (JH), HL81397 (KB), HL151391 (YT), HL137647 (YT), and HL139675 (YY)], NIH/NIA [grant number AG061586 (YT)], NIH/NINDS [grant number NS79353 (YY)], the Presidential Early Career Award for Scientists and Engineers [grant number N00014-16-1-2997 (DD)], and the American Heart Association [Student Scholarship in Cardiovascular Disease (SI) and Howard S. Silverman Scholarship (SI)].

## Conflict of Interest

The authors declare that the research was conducted in the absence of any commercial or financial relationships that could be construed as a potential conflict of interest.

## Publisher’s Note

All claims expressed in this article are solely those of the authors and do not necessarily represent those of their affiliated organizations, or those of the publisher, the editors and the reviewers. Any product that may be evaluated in this article, or claim that may be made by its manufacturer, is not guaranteed or endorsed by the publisher.

## Abbreviations

BMP: Bone morphogenetic protein
CAVD: Calcific aortic valve disease
CHD: Congenital heart disease
CTD: Conotruncal defect
CVD: Cardiovascular diseases
D-flow: disturbed flow
ECM: Extracellular matrix
ECs: Endothelial cells
EFE: Endocardial fibroelastosis
EMT: Epithelial-to-mesenchymal transition
EndMA: Endothelial-to-mesenchymal activation
EndMT: Endothelial-to-mesenchymal transition
GAGs: Glycosaminoglycans
HAECs: Human aortic endothelial cells
HUVECs: Human umbilical vein endothelial cells
IMR: Ischemic mitral regurgitation
MI: Myocardial infarction
MMP: Matrix metalloproteinase
MSC: Mesenchymal stem cell
OFT: Outflow tract
OSS: Oscillatory shear stress
PA: Polyacrylamide
PAH: Pulmonary arterial hypertension
PSS: Pulsatile shear stress
S-flow: Stable flow
SSS: Steady shear stress
TGFβ: Transforming growth factor β
VB: Ventriculobulbar
VE-Cadherin: Vascular endothelial cadherin
VECs: Valvular endothelial cells
VICs: Valvular interstitial cells
